# Comparison of clastogen-induced gene expression profiles in wild-type and DNA repair-deficient *Rad54/Rad54B *cells

**DOI:** 10.1186/1471-2164-11-24

**Published:** 2010-01-12

**Authors:** Anuska G Mahabir, Mirjam M Schaap, Jeroen LA Pennings, Jan van Benthem, Coenraad FM Hendriksen, Harry van Steeg

**Affiliations:** 1 National Institute for Public Health and the Environment (RIVM), Laboratory for Health Protection Research (GBO), P.O.Box 1, NL-3720 BA Bilthoven, the Netherlands; 2 Netherlands Vaccine Institute (NVI), P.O.Box 457, NL-3720 AL Bilthoven, the Netherlands; 3 Netherlands Centre Alternatives to Animal Use (NCA), P.O. Box 80.166, NL-3508 TD Utrecht, the Netherlands

## Abstract

**Background:**

Previously we found that *Rad54/Rad54B *cells are more sensitive towards mitomycin C (MMC) as compared to wild-type (WT) cells. This difference in sensitivity was absent upon exposure to other clastogens like bleomycin (BLM) and γ-radiation. In order to get further insight into possible underlying mechanisms, gene expression changes in WT and *Rad54/Rad54B *MEFs (mouse embryonic fibroblasts) after exposure to the clastogens MMC and BLM were investigated. Exposures of these cells to mutagens (N-ac-AAF and ENU) and vehicle were taken as controls.

**Results:**

Most exposures resulted in an induction of DNA damage signaling and apoptosis genes and a reduced expression of cell division genes in cells of both genotypes. As expected, responses to N-ac-AAF were very similar in both genotypes. ENU exposure did not lead to significant gene expression changes in cells of both genotypes, presumably due to its short half-life. Gene expression responses to clastogens, however, showed a genotype-dependent effect for BLM and MMC. MMC treated *Rad54/Rad54B *MEFs showed no induction of p53-signaling, DNA damage response and apoptosis as seen for all the other treatments.

**Conclusion:**

These data support our finding that different types of clastogens exist and that responses to these types depend on the DNA repair status of the cells.

## Background

DNA double-strand breaks (DSBs) have detrimental effects on the integrity of chromosomes and cell viability. Unrepaired or incorrectly repaired DSBs can lead to loss of chromosomes or cell cycle arrest which may lead to uncontrolled cell growth, cell death or carcinogenesis [1,2]. DSBs mainly arise through exogenous DNA-damaging agents (clastogens) and endogenous sources. Clastogens can be divided into compounds that induce single/double-strand breaks, like bleomycin (BLM) and γ-radiation, and compounds that induce interstrand crosslinks (ICLs), like mitomycin C (MMC). The latter are extremely cytotoxic [3].

The clastogenic potential of chemicals can be tested with different types of genotoxicity assays. In previous studies we measured the *lacZ *mutant frequencies (*lacZ *MF) in both wild-type (WT) and DNA repair-deficient *Rad54/Rad54B *MEFs derived from mice carrying the *lacZ *gene in a plasmid vector. Cells were treated with both mutagenic (causing gene mutations) and clastogenic (causing chromosome aberrations) compounds [4]. The *Rad54/Rad54B *MEFs have a defect in the *Rad54 *and the *Rad54B *genes (both involved in Homologous Recombination (HR) repair), which we assume may cause a shift in the repair of single- or double-strand breaks from HR repair towards non-homologous end-joining (NHEJ) repair, which is an error-prone repair system. This presumed shift between repair systems might cause an accumulation of chromosomal damage induced by clastogens. Since *Rad54/Rad54B *cells have a defect in HR repair, it is to be expected that upon clastogen exposure these cells will accumulate higher *lacZ *mutant frequencies (MF) as compared to WT cells.

It was shown that MEFs isolated from both WT as well as *Rad54/Rad54B *MEFs were able to detect gene mutations and chromosomal aberrations. Surprisingly, of the clastogens used (BLM and MMC), only MMC showed a genotype-dependent effect; *Rad54/Rad54B *MEFs were more sensitive towards MMC treatment as compared to the WT MEFs [4]. Bleomycin (BLM) induced DNA lesions which could be repaired equally effective in cells without active HR repair compared to WT cells, whereas mitomycin C (MMC) showed a differential effect in repair-deficient cells compared to WT cells. This confirms the difference in DNA damage caused by the clastogens, chromosomal breaks (BLM) versus cross linking (MMC).

As different types of clastogens result in genotype-dependent differences in genotoxic sensitivity, we hypothesized that these compounds also trigger separate pathways of (geno)toxicity in the two genotypes. Hence, we further investigated whether different clastogens also led to specific different changes in gene expression patterns upon exposure, and thus if indeed different types of clastogens exist. To this end, we performed microarray analysis with WT and *Rad54/Rad54B *MEFs treated with the clastogens: MMC and BLM, and the mutagens: *N*-acetoxy-2-acetylaminofluorene (N-ac-AAF) and *N*-ethyl-*N*-nitrosourea (ENU). The two mutagens were included as controls aimed at distinguishing general genotoxicity responses as well as genotype-independent responses specific to either clastogens or mutagens. The outcome of our studies confirms our hypothesis that different clastogens lead to specific gene expression changes and moreover the responses are genotype specific.

## Methods

### Isolation of mouse embryonic fibroblasts (MEFs)

Embryos of 13.5 days were harvested from wild-type (WT) and *Rad54/Rad54B *repair-deficient (*Rad54/Rad54B*) mice. All mice were in a C57/BL6 genetic background and were bred and maintained under specific pathogen-free conditions at the animal facility of the Netherlands Vaccine Institute (NVI, Bilthoven, The Netherlands). All animal experiments were approved by the Institute's Animal Ethics Committee and were carried out in accordance with Dutch and international legislation. The liver and head were discarded from the embryonic body (to avoid disturbance during fibroblast growth). The remainder of the embryonic body was trypsinised and cultured in a 75 cm^2 ^flask containing 15 ml culture medium (Dulbecco's Modified Eagle Medium (DMEM) completed with 1% Modified Eagles Medium Non-Essential Amino Acids (MEM NEAA), 1% Penicillin-Streptomycin (PS) and 10% Fetal Bovine Serum (FBS)) at 37°C in a humidified atmosphere containing 3% O_2 _and 10% CO_2 _for 3 days.

After 3 days, the cells were trypsinised, equally divided over two 175 cm^2 ^culture flasks, and were grown for another 4 days. Thereafter, the cells were collected using trypsin and counted in a Bürker-Türk. After centrifugation at 1200 rpm and 4°C for 5 minutes, the cell pellet was resuspended in freezing medium (DMEM completed with 20% FBS. 10% dimethylsulfoxide (DMSO), 1% MEM NEAA and 1% PS) at a concentration of 3 × 10^6 ^cells per ml while keeping it on ice. One ml portions were kept at -80°C for at least 24 hours and were then stored in liquid nitrogen.

### Treatment of MEFs

For each compound and each assay, aliquots of 3 × 10^6 ^cells of both genotypes were seeded and cultured in a 175 cm^2 ^flask containing 30 ml culture medium. Twenty-four hours before treatment, the cells were dissociated with trypsin and cultured into petridishes containing 1 × 10^6 ^cells and a final volume of 10 ml culture medium.

On the day of treatment, the cells were washed once with D-PBS (Dulbecco's phosphate-buffered saline, containing KCl, KH_2_PO_4_, NaCl, Na_2_HPO_4_, without calcium and magnesium) before treatment with the various genotoxic compounds (see Table 1). Untreated MEFs were used as a control. The concentration of each compound was chosen from previously obtained survival data of both WT as well as *Rad54/Rad54B *MEFs treated with the different compounds [4]. In the XTT test, these concentrations resulted in approximately 80% survival.

**Table 1:** Concentrations of all compounds used for the microarray experiment.

Compound	Concentration	Solvent	CAS number
MMC (Mitomycin C)	0.2 μg/ml	PBS	50-07-7

BLM (Bleomycin)	20 μg/ml	PBS	9041-93-4

N-ac-AAF (*N*-acetoxy-2-acetylaminofluorene)	30 μM	DMSO	6098-44-8

ENU (*N*-ethyl-*N*-nitrosourea)	4 mM	Culture medium	759-73-9

The compounds were dissolved in the appropriate solvent (see Table 1) on the day of treatment. The cells were treated with the different compounds in a final volume of 4 ml for 3 hours. After treatment, the cells were washed once with D-PBS and cultured in 10 ml culture medium for an additional 5 hours before collecting them for RNA isolation. Thereafter, the cells were washed once with D-PBS. The cells were dissociated with 750 μl RLT-buffer using a cell scraper and were collected in a 2 ml tube and stored overnight at -80°C until RNA isolation and microarray analysis was performed.

### RNA isolation

Total RNA from each sample was extracted using the Rneasy Mini kit (Qiagen, Valencia, CA, USA), followed by a DNase treatment with RNase-Free DNase Set (Qiagen, Valencia, CA, USA). The RNA concentration was measured using a Nanodrop ND-1000 spectrophotometer (NanoDrop Technologies Inc., Wilmington, DE, USA) and RNA quality was determined on an Agilent 2100 BioAnalyzer (Agilent Technologies, Palo Alto, CA, USA). Measurements were performed according to the manufacturer's protocols. Total RNA samples with an RNA integrity number (RIN) > 7 were used for further analysis.

### Microarray analysis

Mouse oligonucleotide libraries were obtained from Sigma-Compugen Incorporated. The libraries represent a total of 21,825 LEADS™ clusters plus 231 controls. The oligonucleotide libraries and additional control oligos from the Lucidea™ Microarray ScoreCard™ (GE Healthcare) were printed with a Lucidea Spotter (Amersham Pharmacia Biosciences, Piscataway, NJ, USA) on UltraGAPS slides (amino-silane-coated slides, Corning #40017, Corning Life Sciences, Lowell, MA, USA) and processed according to the manufacturer's instructions.

Mouse genome microarrays were used in the analysis of gene expression profiles of WT and *Rad54/Rad54B *MEFs exposed to mutagens and clastogens (see Table 1 in the previous section). For each exposure or control group, five RNA samples were analyzed, each of which was hybridized to an individual microarray slide. In short, Cy3 and Cy5 labeled cRNA samples were prepared as described in the Amino Allyl MessageAmp aRNA kit (Ambion, Austin, Texas, USA) using 1 μg of purified total RNA and a 100 fold dilution of Lucidea Spike-ins (GE Healthcare) as template for the reaction. Test samples were labeled with Cy3 and the common reference was labeled with Cy5. The common reference was made by combining 1 μg of each test sample, labeling 1 μg portions of this pool with Cy5 and pooling the resulting Cy5 labeled material afterwards. 1 μg of Cy3 cRNA was combined with 1 μg Cy5 labeled cRNA and incubated for 30 minutes at 60°C in the presence of fragmentation buffer (Agilent). The fragmentized solution was mixed in a 1:1 ratio with a 2 × hybridization buffer (Agilent) and transferred to the microarray. A sandwich of the microarray with a backing slide was hybridized overnight in a Surehyb chamber. Hybridization was performed overnight according to the Agilent, Low NA input Linear Amplification procedure at 65°C. The Surehyb chamber was disassembled in GE wash buffer 1 and washed for 1 minute at room temperature and for another minute in GE wash buffer 2 at 37°C. Microarrays were quickly dried by dipping in isopropanol and a short spin at 230 rcf. Arrays were scanned at two wavelengths (Cy3 and Cy5, or 532 and 633 nm, respectively) on an Agilent G2565 microarray scanner.

### Data analysis and statistics

Array Vision software (Imaging Research, St. Catherine's, Ontario, Canada) was used to determine median Cy3 and Cy5 signal intensities for each separate spot and background noise. Quality control was performed on raw data by means of visual inspection of the scanned images, as well as a check on the scatter and MA (ratio-intensity) plots. The control spots present on the slide were used for quality control, but excluded from the further analysis. Raw signal data for oligonucleotide-containing spots were normalized with R software http://www.r-project.org by using a three-step approach [5] that consisted of (1) natural log transformation, (2) quantile normalization of all scans, (3) correction of the sample spot signal for the corresponding reference spot signal. Raw and normalized data are available at ArrayExpress http://www.ebi.ac.uk/arrayexpress under accession number E-TABM-786.

Differences in gene expression between experimental MEFs sample groups were determined by a one-way ANOVA. Genes with a False Discovery Rate (FDR) < 0.05 and a Fold Ratio (FR) > 1.5 (between treated and control group) were considered differently expressed. Correlations between sets of differently expressed genes were calculated for the union of the two sets. Enrichment for Gene Ontology and other functional terms was determined by DAVID/EASE http://david.abcc.ncifcrf.gov[6]. Additional analyses were performed using GeneMaths (Applied Maths, St-Martens-Latem, Belgium). Principal Component Analysis (PCA) was performed on the average whole-genome gene expression profile of the experimental groups. For clustering analysis, the merged data of all genes regulated in at least one treatment-control comparison were combined into a table with the appropriate ln-ratio/control values, which was further analyzed using Euclidean distance and Ward linkage.

Groupwise regulation of Gene Ontology categories and gene sets were determined by Gene Set Enrichment Analysis (GSEA) [7] using the pre-ranked GSEA option under default analysis parameters. Gene set collections used were the c5 (Gene Ontology) and c2 (expert curated) gene sets provide by MsigDB http://www.broad.mit.edu/gsea/msigdb/, and additional gene set collections developed in house. Gene sets were considered regulated if the GSEA *p*-value was < 0.05 and the FDR was < 0.25.

## Results

### Basal genotype gene expression differences

**Figure 1:**
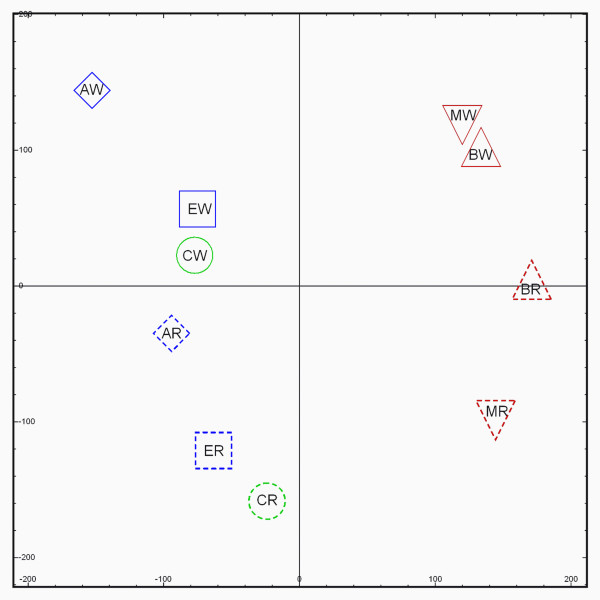
**Principal component analysis on genome-wide expression profiles for the experimental groups**. **CW**: WT MEFs - untreated, **CR**: *Rad54/Rad54B *MEFs - untreated, (circle); **AW**: WT MEFs - N-ac-AAF, **AR**: *Rad54/Rad54B *MEFs - N-ac-AAF, (diamond); **EW**: WT MEFs - ENU, **ER**: *Rad54/Rad54B *MEFs - ENU, (square); **BW**: WT MEFs - BLM, **BR**: *Rad54/Rad54B *MEFs - BLM, (triangle); **MW**: WT MEFs - MMC, **MR**: *Rad54/Rad54B *MEFs - MMC (inverted triangle).

After microarray data normalization, Principal Component Analysis was performed on the average gene expression profiles of the experimental groups (Figure 1). This indicates a separation between all exposed and control WT versus *Rad54/Rad54B *MEFs, respectively. However, in contrast to the relatively large genotype difference suggested by the PCA, only 12 genes were found to be differentially expressed between these groups (FDR < 5%, FR > 1.5). GSEA analysis showed a subtle but coordinate relative down-regulation of cell division related genes in the *Rad54/Rad54B *MEFs compared to the WT MEFs. Likewise, this genotype showed a similarly subtle but coordinate trend in up-regulation of immunological pathways and immune-cell associated genes as compared to the WT MEFs. This indicates that the genotype separation indicated by the PCA is due to small changes in the activity of the cell division machinery and a higher percentage of (precursors of) immune cells. It should be mentioned here that MEFs are derived from a large part of the embryo and consist of a mixed cell population, containing mostly fibroblast but also other cell types. Further GSEA analysis using cell type-specific gene sets could not ascribe the shift in immune cell expression to specific immunological cell lineages.

### Gene expression response to mutagens

**Figure 2:**
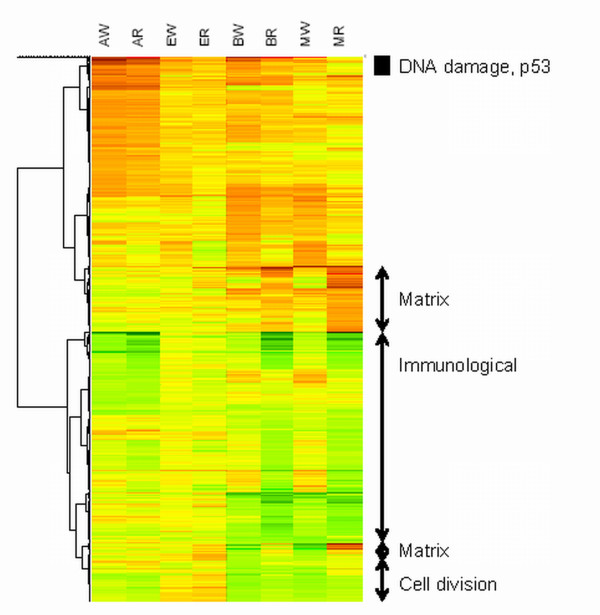
**Hierarchical clustering of gene expression changes in WT and *Rad54/Rad54B *MEFs after mutagen or clastogen exposure**. Expression changes are given relative to the corresponding control, where red represents up-regulation, green down-regulation and yellow no difference in gene expression levels. The black bar indicates the cluster showing the strongest genotype-dependent effect for the MMC-exposed MEFs. Exposures are as follows: **AW**: WT MEFs - N-ac-AAF, **AR**: *Rad54/Rad54B *MEFs - N-ac-AAF, **EW**: WT MEFs - ENU, **ER**: *Rad54/Rad54B *MEFs - ENU, **BW**: WT MEFs - BLM, **BR**: *Rad54/Rad54B *MEFs - BLM, **MW**: WT MEFs - MMC, **MR**: *Rad54/Rad54B *MEFs - MMC.

Exposure to N-ac-AAF resulted in 150 and 143 differently expressed genes in WT and *Rad54/Rad54B *MEFs, respectively. The responses were highly comparable (R = 0.96) for both genotypes, as can also be seen from Figure 2. Functional annotation showed that the strongest induced genes were involved in a number of connected pathways such as p53-signaling, DNA damage response, and apoptosis (*Pmaip1*, *Ccng1*, *Btg2*, *Mdm2*, *Cdkn1a*, *Rprm*, *Perp, Bax*). In addition, there was up-regulation for a number of oxidative stress genes (*Mt1*, *Gsta1*, *Gsta2*, *Gsta4*, *Gclm*). Down-regulation was mainly observed for cell division genes (e.g. *Ccnb1*, *Ccnb2*, *Ccna2*, *Aurka*).

ENU exposure did not result in any differently expressed genes in WT and only 2 in *Rad54/Rad54B *MEFs. Although hardly any genes were significantly regulated, we did observe that the DNA damage responsive genes regulated by N-ac-AAF showed a similar but weaker trend upon ENU exposure (Figure 2 and Table 2).

**Table 2:** Gene expression changes for DNA damage response and cell cycle genes in WT and *Rad54/Rad54B *MEFs after exposure to mutagens and clastogens.

Gene symbol	Alias	WT^**1 **^- N-ac-AAF	Rad^**2 **^- N-ac-AAF	WT - ENU	Rad - ENU	WT - BLM	Rad - BLM	WT - MMC	Rad - MMC
Pmaip1	Noxa	3.26*	2.40	1.39	1.29	4.12*	2.86*	2.26	-1.02

Ccng1		2.74*	2.00*	1.77	1.28	2.99*	2.13*	1.54	-1.11

Btg2		2.46*	1.68*	1.59	1.09	2.77*	1.75*	1.55	1.13

Mdm2		6.37*	3.40*	2.21	1.54	3.08*	1.95*	1.99	1.08

Cdkn1a	P21	4.20*	2.81*	2.09	1.54	3.06*	2.00*	1.67	1.35

Rprm		2.41*	2.37*	1.21	1.31	2.50*	1.97*	1.14	1.38

Perp		1.54*	1.40	1.07	1.08	1.48	1.46	1.05	1.08

Bax		1.55*	1.52*	1.14	1.29	1.50	1.40	1.25	-1.05

Bbc3	Puma	1.34	1.24	1.16	1.10	1.54*	1.33	1.41	1.11

Mgmt		1.01	1.06	1.15	1.16	1.72*	1.29	1.07	-1.08

Ccnb1		-1.63*	-1.78*	1.07	1.20	-1.6*	-1.43	-1.58	-1.61

Ccnb2		-1.64*	-1.56*	-1.08	1.16	-1.67*	-1.48	-1.79*	-1.42

Plk1		-1.55	-1.72*	1.07	1.19	-2.00*	-1.57*	-2.04*	-1.51

Ccna2		-1.24	-1.37	1.07	1.16	-2.01*	-1.43	-1.58*	-1.38

Ube2c		-1.25	-1.48	1.25	1.23	-1.44	-1.42	-1.70*	-1.58

Aurka		-1.28	-1.59*	1.29	1.22	-1.81*	-1.50*	-1.71*	-1.58*

Cdkn3		-1.44	-1.45	-1.01	1.16	-1.48	-1.22	-1.53*	-1.24

Cdca3		-1.31	-1.39	-1.00	1.17	-1.51*	-1.32	-1.44	-1.38

Cdc2a		-1.16	-1.22	1.18	1.27	-1.64*	-1.33	-1.56*	-1.44

Bub1b		-1.10	-1.31	1.10	1.07	-1.67*	-1.43	-1.49	-1.27

Cdca8		-1.12	-1.29	1.17	1.11	-1.57*	-1.32	-1.34	-1.35

Cdkn2d	P19	-1.24	-1.17	1.10	1.45	-1.47	-1.26	-1.65*	-1.04

Cdkn2c	P18	1.05	-1.05	1.19	1.42	-1.59*	-1.34	-1.60*	-1.09

^1^WT are wild-type MEFs treated with the different compounds

^2^Rad are *Rad54/Rad54B *MEFs treated with the different compounds

*An asterisk indicates a significant regulated gene (at FDR < 5% and ratio > ± 1.5)

### Gene expression response to clastogens

The numbers of differently expressed genes in WT and *Rad54/Rad54B *MEFs after BLM treatment were 166 and 178, respectively. Responses for both genotypes were comparable (R = 0.75), but more different than those observed for the mutagen exposures (see above). Functional annotation of differentially expressed genes showed the predominant effect was induction of genes involved in p53-signaling, DNA damage response, and apoptosis (*Pmaip1*, *Ccng1*, *Btg2*, *Mdm2*, *Cdkn1a*, *Rprm, Bbc3*). Down-regulation was observed for several cell division genes (e.g. *Ccnb1*, *Ccnb2*, *Ccna2*, *Aurka*, *Cdca3*, *Cdc2a*, *Bub1b*, *Cdca8*). These changes were overall similar to those observed for the mutagen N-ac-AAF.

For MMC treatment, the numbers of differentially expressed genes were 76 and 156 for WT and *Rad54/Rad54B *MEFs, respectively. Here, the responses were different (R = 0.29) between the MEFs of the two genotypes. For the WT MEFs, functional annotation showed induction of p53-signaling and DNA damage response genes (*Pmaip1*, *Ccng1*, *Btg2*, *Mdm2*, *Cdkn1a*), and down-regulation of cell division (*Ccnb1*, *Ccnb2*, *Ccna2*, *Ube2c*, *Aurka*, *Cdc2a*) genes, in a similar manner as seen for BLM and N-ac-AAF. For the *Rad54/Rad54B *MEFs, we observed down-regulation of cell division genes similar to that found in the corresponding WT exposure. However, induction of p53-signaling, DNA damage response, and apoptosis were not found in the MMC-treated *Rad54/Rad54B *MEFs. Comparing these responses among the clastogen-exposed groups showed that these were almost absent in the MMC-treated *Rad54/Rad54B *MEFs but present in the other groups (Figure 2 (indicated as a black block) and Table 2). This latter finding was confirmed in the GSEA results: the canonical p53-related pathways in the MsigDB C2 database all showed a significant pathway-level induction for the different treatment vs control comparisons; except for the MMC exposed *Rad54/Rad54B *MEFs where p values as well as the corresponding FDR were > 0.25.

### Gene expression response comparison clastogens to mutagens

Comparing the gene expression changes for mutagen and clastogen exposed MEFs showed a cluster of commonly regulated genes for all the exposures except for the MMC-treated *Rad54/Rad54B *MEFs. This cluster is indicated in Figure 2 with a black bar. Additionally, for both BLM and MMC exposed MEFS, gene expression down-regulations were found that were not (or less) present in mutagen exposed MEFs of both genotypes (Figure 2). Functional annotation showed that the majority of the genes involved are either involved in immunological pathways or known immune cell markers. Interestingly, these genes show a stronger down-regulation in the *Rad54/Rad54B *than in the WT MEFs, indicating that this phenomenon is linked to the higher proportion of immune cells in the *Rad54/Rad54B *MEFs. Clustering also revealed an increase in extracellular matrix gene expression in the clastogen treated MEFs, which was very pronounced in the *Rad54/Rad54B *MEFs but less induced or even down-regulated in WT MEFs (Figure 2). As extracellular matrix genes are highly expressed in fibroblast this indicates a relative enrichment for fibroblast mRNA when compared to the down-regulation of immune cell mRNA in the total mRNA fraction.

## Discussion

We have previously demonstrated that WT and *Rad54/Rad54B *MEFs were able to detect both mutagen and clastogen activity using *lacZ *as a reporter gene. For the MMC-exposed, but not the BLM-exposed MEFs, we observed an increase in *lacZ *mutant frequency in the *Rad54/Rad54B *MEFs compared to the WT MEFs. This confirms that different types of clastogens exist, which cause genotype-dependent differences in genetic damage. To investigate whether these differences are reflected in triggering differences in genotoxicity response pathways, we examined gene expression changes in WT and *Rad54/Rad54B *MEFs upon exposure to two types of clastogens, two mutagens and unexposed controls.

PCA analysis shows that an overall genotype-dependent difference exists between all WT versus all *Rad54/Rad54B *MEFs. A combination of statistical analysis at the gene expression level and a threshold-free whole-genome analysis (GSEA) showed that these differences could be ascribed to a combination of lower activity of cell division genes and an increase in the proportion of immune cells in the *Rad54/Rad54B *MEFs compared to the WT MEFs. The presence of immune cells among mouse embryonic fibroblast cells can be explained because MEFs are not exclusively derived from fibroblast containing tissues. Instead, MEFs originate from a larger part of the embryo, excluding liver and head but including immunologically relevant tissues. By the choice of culturing conditions, further MEF culture selects for fibroblast cells. However, this selection will not be complete and some immune cells remained present in the MEFs used for the experiments, especially those described for *Rad54/Rad54B *MEFs.

PCA visualization of the whole-genome data (Figure 1) shows a similar shift in both direction and length for the gene expression profiles for each compound compared to their respective control. In the case of ENU exposed MEFs the overall effect is small and for these exposures hardly any significantly regulated genes were found in MEFs of both genotypes. Clastogen-exposed MEFs (both MMC and BLM) show an overall similar trend in the PCA and were different for the N-ac-AAF-exposed MEFs, indicating that there is a difference in gene expression response after mutagens and clastogens exposure.

For most of the exposures, a broad-scale DNA damage response was observed. This included genes that are involved in apoptosis (*Pmaip1*, *Mdm2, Cdkn1a*, *Perp*, *Bax*, *Bbc3*), cell cycle arrest (*Ccng1*, *Btg2*, *Mdm2, Cdkn1a*, *Rprm*), and DNA repair (*Mgmt*); with a role for p53 in their activation being a common factor. Responses in these genes lead to temporarily cell cycle arrest and DNA repair or apoptosis. In line with this finding, we also observed a down-regulation of cell division genes for most of the exposures. Induction of oxidative stress genes was only found for the N-ac-AAF exposed cells by means of induction of several GST enzymes. Although oxidative stress plays a role in inducing genotoxicity, the response found here can also be part of a general protection mechanism against this compound. Exposure to clastogens, but not mutagens, resulted in a relative down-regulation of immune cell-associated genes and a relative up-regulation of fibroblast-associated genes. Both these effects were more pronounced in the *Rad54/Rad54B *MEFs as compared to the WT MEFs. The relative down-regulation of immune cell-associated genes suggests that immune cells and their precursors are more sensitive to clastogen exposure than to mutagen exposure. The higher proportion of immunological cells in the *Rad54/Rad54B *MEFs population provides an explanation for the enhanced effects observed for this genotype. The relative up-regulation of extracellular matrix genes can be explained in that a lower presence of immune cell-derived mRNA in the total mRNA fraction leads to a relative increase for mRNA highly expressed in other (e.g. fibroblast) cells. This is consistent with the finding that this effect is also more pronounced in the *Rad54/Rad54B *genotype than WT MEFs. It should be noted that the differences in cell composition, and therefore transcriptional changes in lineage specific genes upon treatment, are not necessarily related to genotype-dependent differences between WT and *Rad54/Rad54B *MEFs. As MEFs are not clonal in their origin, some variations in cell composition between isolations are inherent to the use of such cells. Transcriptional changes in cell lineage specific genes reflect the natural variation between MEF batches coinciding with different sensitivities between cell lineages to different (classes of) genotoxic compounds.

The concentrations used in the experiment were chosen to result in comparable effects on survival and indeed the WT response for N-ac-AAF, BLM, and MMC was found to give similar degrees of gene expression changes. In contrast, ENU gave much weaker gene expression changes in both MEF genotypes. A possible explanation for this could be the half life of ENU which is approximately 1 hour in culture medium [8]. The gene expression changes were measured 8 hours after treatment with the different compounds. Since ENU only has a half-life of 1 hour, most of its reactivity will have disappeared within the first few hours after exposure. The remaining time gives the cells the ability to restore the DNA damage caused by ENU exposure. Therefore, there will be no or a very low effect measured of the ENU exposure 8 hours after treatment. This was seen as both ENU treated as well as untreated samples showed quite similar gene expression changes in both WT as well as *Rad54/Rad54B *MEFs.

Effects for the N-ac-AAF treatments in WT and *Rad54/Rad54B *MEFs were similar in their response, and the response for ENU treatment was virtually absent in MEFs of both genotypes. Therefore, it can be said that mutagens give the same gene expression response in both WT and *Rad54/Rad54B *MEFs. For the clastogen treatments, however, this was not the case. For the BLM treatment, the response was moderately similar, albeit comparable (R = 0.75) for MEFs of both genotypes. An even stronger difference was found for after MMC treatment (R = 0.29) between MEFs of both genotypes. Thus, the response for *Rad54/Rad54B *MEFs is different from the WT response to clastogens, making it (at least partially) clastogen specific. Remarkably, in addition to differences in gene expression profiles after clastogen and mutagen treatment, there is also a difference between the two clastogens used (BLM and MMC). The major difference between the responses to BLM and MMC lies in a weaker response through the p53-signaling pathway upon MMC exposure of *Rad54/Rad54B *MEFs, whereas the reduction in cell division genes was not affected.

The reduction in p53-signaling pathway genes for MMC, though not for BLM, is in line with our previous finding that two types of clastogens exist [4]. One including BLM and γ-radiation, acting mainly through single- and/or double-strand breaks, that can be repaired equally effective in cells with and without active HR repair. MMC on the other hand belongs to a class that causes DNA crosslinks which show a differential effect in WT versus HR-repair-deficient cells. All these findings indicate that MMC damage repair is HR dependent. In this study, the MMC response deviates between WT and *Rad54/Rad54B *MEFs compared to the BLM response. The reduced p53-signaling after MMC exposure in *Rad54/Rad54B *MEFs could provide a mechanistic explanation for the increased *lacZ *MF after MMC exposure [4], as an impaired DNA damage response will lead to a weaker DNA repair response and therefore a larger percentage of the cells will carry a *lacZ *mutation.

This study provides evidence that the difference in DNA damage response between BLM and MMC is caused by insufficient p53-signaling at the gene expression level, presumably due to lack of DNA crosslink damage recognition in MMC-exposed *Rad54/Rad54B *MEFs. This suggests that HR is not only necessary for crosslink repair, but that the *Rad54 *and/or *Rad54B *genes are involved in DNA crosslink damage recognition.

## Conclusions

In this study, we used WT and DNA repair deficient *Rad54/Rad54B *MEFs to study transcriptional responses to two different clastogens; bleomycin (BLM), which causes chromosomal breaks, and the crosslinking agent mitomycin C (MMC). The mutagens N-acetoxy-2-acetylaminofluorene (N-ac-AAF) and N-ethyl-N-nitrosourea (ENU) induced similar gene expression changes in MEFs of both genotypes, however, the two clastogens triggered different responses. In MMC-exposed *Rad54/Rad54B *MEFs we could not detect a p53-dependent response to genotoxic offense, which was as expected normally induced in MMC-exposed WT MEFs as well as in BLM-exposed MEFs being either repair deficient or proficient. This study is in line with our previous study [4] and lends further support to our hypothesis that different types of clastogens exist. This can have implications for compound hazard identification.

## Competing interests

The authors declare that they have no competing interests.

## Authors' contributions

AGM carried out the experiments and wrote the manuscript. MMS provided technical support. JLAP analysed the data and helped write the paper. JvB and CFMH supervised the studies. HvS designed the study, helped interpret the data, and revised the manuscript. All authors read and approved the final manuscript.
